# Machine Learning-Based Prediction of IVF Outcomes: The Central Role of Female Preprocedural Factors

**DOI:** 10.3390/biomedicines13112768

**Published:** 2025-11-12

**Authors:** Kristóf Bereczki, Mátyás Bukva, Viktor Vedelek, Bernadett Nádasdi, Zoltán Kozinszky, Rita Sinka, Csaba Bereczki, Anna Vágvölgyi, János Zádori

**Affiliations:** 1 Department of Obstetrics and Gynecology, Albert Szent-Györgyi Medical School, University of Szeged, H-6725 Szeged, Hungary; bereczkikristof9@gmail.com (K.B.); kozinszky@gmail.com (Z.K.); zadori.janos@med.u-szeged.hu (J.Z.); 2 Department of Immunology, Albert Szent-Györgyi Medical School, Faculty of Science and Informatics, University of Szeged, H-6720 Szeged, Hungary; 3 Department of Pediatrics and Pediatric Health Center, University of Szeged Albert Szent-Györgyi Health Center, H-6725 Szeged, Hungary; bereczki.csaba@med.u-szeged.hu; 4 Laboratory of Microscopic Image Analysis and Machine Learning, Institute of Biochemistry, Biological Research Centre, Hungarian Research Network (HUN-REN), H-6726 Szeged, Hungary; 5 Department of Genetics, Faculty of Science and Informatics, University of Szeged, H-6726 Szeged, Hungaryrsinka@bio.u-szeged.hu (R.S.); 6 Department of Medicine, Albert Szent-Györgyi Medical School, University of Szeged, H-6725 Szeged, Hungary; nadasdi.bernadett@med.u-szeged.hu (B.N.);; 7 Capio Specialized Center for Gynecology, 182 88, Stockholm, Sweden; 8 Institute of Reproductive Medicine, Albert Szent-Gyorgyi Medical School, University of Szeged, H-6720 Szeged, Hungary

**Keywords:** preprocedural factors, in vitro fertilization, clinical pregnancy, live birth, machine learning

## Abstract

**Objectives:** We aimed to develop and validate a per-cycle prediction model for in vitro fertilization (IVF) success using only preprocedural clinical variables available at the first consultation. **Methods:** We retrospectively analysed 1243 IVF/ICSI cycles (University of Szeged, 21 January 2022–12 December 2023). An Extreme Gradient Boosting (XGBoost version 1.7.7.1) classifier was trained on 14 baseline predictors (e.g., female age, AMH, BMI, FSH, LH, sperm concentration/motility, and infertility duration). A parsimonious 9-variable model was derived by feature importance. Model performance was assessed on the untouched test set and, as a final step, on an independent same-centre external validation cohort (*n* = 92) without re-fitting or recalibration. **Results:** The 9-variable model achieved an AUC of 0.876 on the internal test set, with an accuracy of 81.70% (95% CI 76.30–86.30%), sensitivity of 75.60%, specificity of 84.40%, PPV of 68.60%, and NPV of 88.50%. In external validation, the model maintained strong performance with an accuracy of 78.30%, confirming consistent discrimination on an independent same-centre cohort. Female age was the dominant high-impact feature, while AMH and BMI acted as “workhorse” predictors, and male factors added incremental value. **Conclusions:** IVF outcome can be predicted at the first visit using routinely collected preprocedural data. The model showed consistent discrimination internally and in external validation, supporting its potential utility for early, individualized counselling and treatment planning.

## 1. Introduction

Over the past decades, in vitro fertilization (IVF) has profoundly transformed reproductive medicine, offering new possibilities for couples and individuals struggling with infertility. Despite continuous improvements in laboratory techniques and clinical protocols, IVF outcomes remain variable, with live birth rates per initiated cycle averaging approximately 20–30% worldwide [1]. This inconsistency has stimulated extensive research into the determinants of IVF success, particularly during the preimplantation stage. The preimplantation period—spanning from fertilization to embryo transfer—represents a critical window characterized by fundamental biological processes, including oocyte quality, sperm function, fertilization dynamics, early embryonic development, and endometrial receptivity. Each of these factors plays a crucial role in determining the likelihood of successful implantation and subsequent pregnancy [2].

The present research focuses on a key direction in modern IVF studies: identifying factors that can predict treatment outcomes before initiation—often termed baseline predictors or preprocedural factors. By integrating demographic, clinical, and initial laboratory data, preprocedural factors allow clinicians to individualize ovarian stimulation, provide tailored counselling, and optimize treatment strategies according to each patient’s fertility profile.

The emergence of machine learning (ML) has introduced a new level of precision to reproductive medicine [3,4,5]. Using large-scale datasets from clinical, imaging, and molecular sources, ML algorithms can uncover complex patterns and predictive markers that are not easily detectable by human assessment. Furthermore, ML applications have been increasingly employed to personalize stimulation regimens, refine endometrial receptivity evaluation, and predict IVF outcomes based on patient-specific features [6]. These developments offer significant potential to improve both the accuracy and efficiency of assisted reproduction.

Within this framework, the present study applies the Extreme Gradient Boosting (XGBoost) algorithm, recognized for its robust performance in modelling intricate, non-linear relationships—an essential feature when analysing multifactorial phenomena such as IVF success [7]. Unlike traditional linear approaches, XGBoost employs ensembles of decision trees to capture complex interactions and threshold effects. Its predictive capacity is further refined through advanced hyperparameter optimization, ensuring a balance between model complexity and generalizability. This makes XGBoost particularly advantageous for handling high-dimensional clinical datasets where both accuracy and interpretability are crucial.

This study seeks to investigate critical preimplantation determinants, with a particular emphasis on preprocedural factors and baseline predictors that influence IVF outcomes. Central to this investigation is the integration of ML methodologies. The objectives of the study are twofold: (1) to identify the most salient preimplantation variables associated with IVF success and (2) to assess the extent to which ML techniques—specifically Extreme Gradient Boosting—can enhance predictive accuracy and inform clinical decision-making.

## 2. Results

### 2.1. Univariate Analysis Highlights Distinct Patterns in IVF Outcomes

Univariate analysis revealed significant differences between successful and unsuccessful IVF cycles for female age, anti-Müllerian hormone (AMH), follicle-stimulating hormone (FSH), and male age (*p* < 0.0001). In successful outcomes, women were younger (median: 34 vs. 37 years), had higher AMH levels (2.1 vs. 1.6 pmol/L), and lower FSH levels (6.7 vs. 7.4 IU/L), as were their male partners (median: 37 vs. 39 years) (Table 1). In contrast, luteinizing hormone (LH), body mass index (BMI), infertility duration, and sperm parameters showed no significant differences (*p* > 0.05). Importantly, the absence of univariate significance does not preclude the relevance of these variables, as traditional tests cannot detect complex relationships (e.g., interactions or non-linear effects). Therefore, an XGBoost model was employed to investigate these higher-order patterns.

### 2.2. The Model Achieves High Accuracy in Outcome Prediction Using All Available Clinical Variables

**Figure 1:**
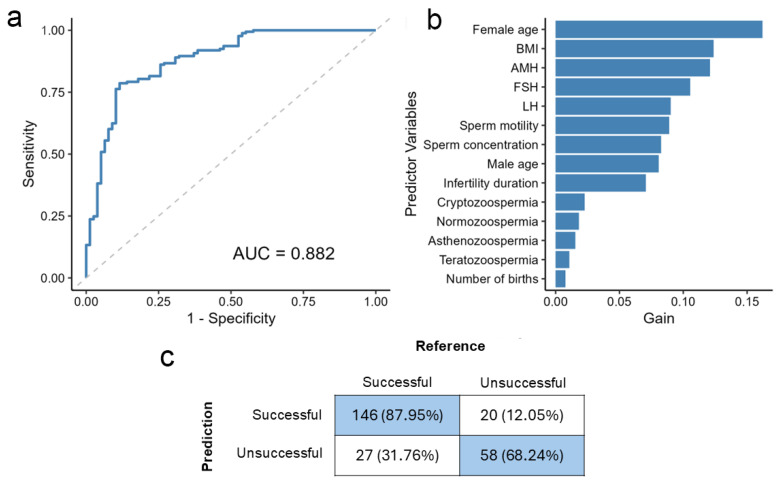
Efficiency of the overall model. (**a**) Receiver operating characteristic (ROC) curve for the full model. (**b**) Bar plot of variable importance based on Gain, showing the contribution of all 14 predictors. (**c**) Confusion matrix of the model, with percentage values representing the row percentages. AMH: anti-Müllerian hormone; AUC: the area under the ROC curve; BMI: body mass index; FSH: follicle-stimulating hormone; LH: luteinizing hormone.

The initial model, developed with all 14 predictors, showed robust performance on the test set, achieving an area under the curve (AUC) of 0.88 (Figure 1a). The model reached an overall accuracy of 81.67% (95% CI: 76.32–86.26%), significantly surpassing the no-information rate (NIR) of 68.92% (*p* < 0.001). It yielded a sensitivity of 75.64%, a specificity of 84.39%, a positive predictive value (PPV) of 68.60%, and a negative predictive value (NPV) of 88.48%. The confusion matrix (Figure 1b) details these classification results.

Feature importance analysis identified female age as the most influential predictor. Predictor importance declined sharply after the ninth variable, infertility duration, suggesting a relevance threshold. In contrast, factors such as sperm type and the number of previous births offered negligible contributions to the model’s predictive power (Figure 1b).

### 2.3. Removing Negligible Variables Balances Simplicity and Performance

To balance predictive power with interpretability, we developed a refined model focused on the top nine predictors identified by the Gain metric: female age, AMH, BMI, FSH, LH, sperm concentration, sperm motility, male age, and infertility duration. This streamlined approach removed five less influential variables without sacrificing performance.

**Figure 2:**
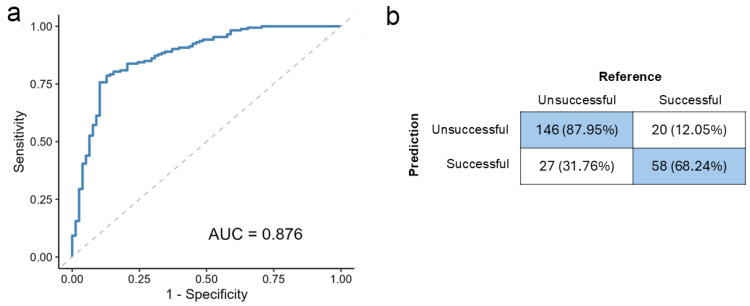
Efficiency of the 9-variable model. (**a**) Receiver operating characteristic (ROC) curve for the 9-variable model. (**b**) Confusion matrix of the model, with percentage values representing the row percentages. AUC: the area under the ROC curve.

On the test data, the 9-feature model achieved an AUC of 0.876, showing nearly identical discriminative power to the full model’s 0.882 (Figure 2a). Its overall accuracy reached 81.67% (95% CI: 76.32–86.26%), significantly exceeding the no-information rate (*p* < 0.001). The model yielded a sensitivity of 75.64%, a specificity of 84.39%, a PPV of 68.60%, and an NPV of 88.48%, leading to a balanced accuracy of 80.02%. Figure 2b presents the detailed confusion matrix.

These results confirm that removing less influential predictors created a more parsimonious model without compromising its robust predictive performance.

### 2.4. Feature Metrics Unveil the Impact of Key Predictors on IVF Outcome Classification

**Figure 3:**
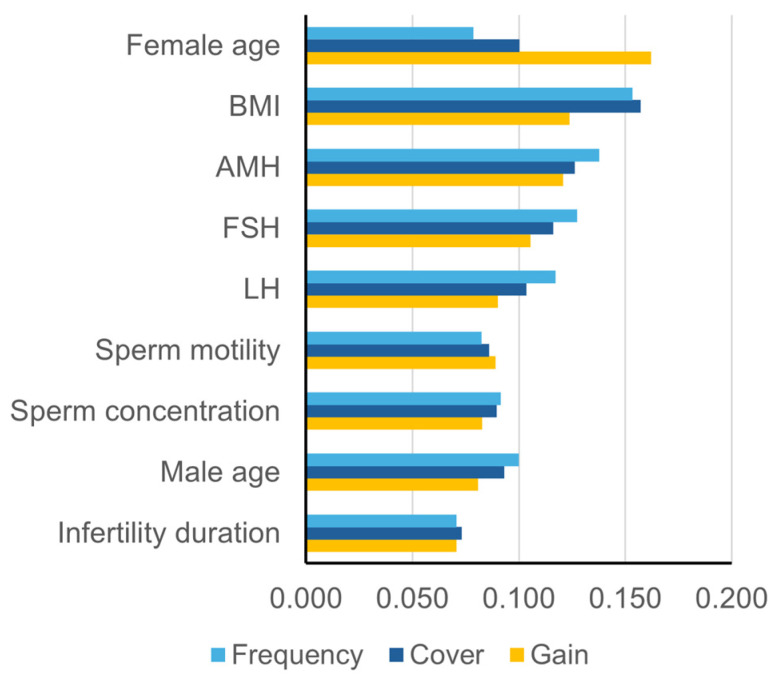
Feature importance metrics in the refined model. This figure presents the Gain, Cover, and Frequency values for the nine key predictors in the refined model. Gain (yellow) quantifies the relative contribution of each variable to classification improvement, Cover (blue) indicates the proportion of data samples affected by splits involving the variable, and Frequency (light blue) represents how often each feature was used in the model. Female age exhibits the highest Gain, while BMI and AMH show greater Cover and Frequency, suggesting their broader influence across multiple decision trees. AMH: anti-Müllerian hormone; BMI: body mass index; FSH: follicle-stimulating hormone; LH: luteinizing hormone.

To understand how each predictor contributed to the refined model, we analysed their Gain, Cover, and Frequency values (Figure 3). These metrics reveal a feature’s impact on accuracy (Gain), the proportion of data it affects (Cover), and how often the model uses it (Frequency).

The analysis identified distinct functional roles. Female age emerged as a “high-impact” feature, providing the largest improvement in accuracy per split with the highest Gain (0.182). Its moderate Cover (0.109) and low Frequency (0.090) suggest the model applies it selectively for decisive classifications.

In contrast, BMI and AMH functioned as “workhorse” predictors. Despite slightly lower Gain values (0.130 and 0.131), their high Frequency and Cover demonstrate their consistent application across the dataset, making them crucial for the model’s fine-tuning process.

The other six predictors—including FSH, sperm motility, and LH—acted in supportive roles. These features exhibited more modest Gain values (ranging from 0.083 to 0.105), offering incremental but valuable improvements that helped hone predictions for specific subgroups. This multi-faceted view of feature importance clarifies how different variables collaborate to achieve high predictive accuracy.

### 2.5. Consistent Performance on an Independent Same-Centre Validation Cohort

As the final step of evaluation, we tested the final nine-variable model (detailed in Section 2.3) on a non-overlapping, same-centre external cohort using the pre-specified parameters, without any re-fitting or re-calibration.

Among the 92 patients included, 63 had unsuccessful and 29 had successful IVF outcomes. The model correctly identified 52 of the unsuccessful and 20 of the successful cases, while 11 unsuccessful and 9 successful cases were misclassified. This corresponded to an overall accuracy of 78.3%, with a sensitivity of 68.9%, specificity of 82.5%, positive predictive value of 69.0%, and negative predictive value of 82.5%. The external validation results closely mirrored the internal hold-out performance (detailed in Section 2.3), supporting the model’s robustness and its practical applicability for first-visit, pre-procedural counselling within the same clinical environment.

## 3. Discussion

Accurate prognosis is a critical determinant in medically assisted reproduction, profoundly shaping the patient experience. The psychological distress and financial strain of the procedure can be significant, and a comprehensive, evidence-based evaluation directly influences patient resilience and dropout rates. Patients often modify their lifestyle and work environments in response to the demands of treatment, highlighting the need for reliable predictive information.

Given that IVF success is influenced by multiple interacting determinants and represents a complex, multifactorial prediction task, Machine Learning (ML) offers a practical route to enhance the accuracy of in vitro fertilization (IVF) outcome predictions and to support personalised treatment planning at the very first consultation, because it can integrate heterogeneous, multidimensional inputs and identify non-linear patterns—capabilities demonstrated broadly beyond IVF [8,9].

In this study, we evaluated an XGBoost model, initially developed with 14 pre-implantation predictors and subsequently refined to the nine most informative variables based on their relative importance: female age, AMH, BMI, FSH, LH, sperm concentration, sperm motility, male age, and infertility duration. Our analysis revealed distinct roles for these predictors.

Female age emerged as the dominant high-impact variable, providing decisive classifications, while AMH and BMI acted as “workhorse” features that contributed frequently across diverse patient profiles, refining risk estimates throughout the decision space. The remaining factors, including FSH, LH, and male-specific variables, offered supportive contributions that improved discrimination in borderline cases.

To align these findings with current clinical guidance, it is important to note that AMH is widely recognised as a sensitive marker of ovarian reserve and a key parameter for individualising ovarian stimulation, yet international guidelines emphasise that it should not be used as a stand-alone fertility test or the sole predictor of live birth—precisely reflecting how our model integrates this marker: as a context-dependent refiner rather than an independent determinant [10,11].

Similarly, consensus statements and large cohort studies consistently associate increasing BMI with reduced clinical pregnancy and live-birth rates in assisted reproductive technology, particularly in autologous cycles, with attenuated effects in donor-oocyte settings. The model’s recurrent use of BMI as a “workhorse” predictor is therefore directionally consistent with clinical evidence and counselling practice [12,13].

A recent study highlighted that while the number of cycles did not affect pregnancy outcomes in young women, it was found to be related to success in older women [14].

The findings from this XGBoost model offer new insights when compared to our previous work, which used a Support Vector Machine (SVM) on a similar patient cohort [15].

That SVM model, which also incorporated post-procedural data like embryo quality, achieved a lower prediction accuracy (61.71%) and identified embryo quality and maternal age as the only strong predictors. Notably, the SVM model did not find significant predictive value in BMI, FSH, or AMH, and the impact of male factors was negligible. The current XGBoost model, however, underscores the considerable predictive value of these hormonal markers and BMI, along with three male factors. This suggests that XGBoost possesses an enhanced capability to capture the complex, non-linear interactions among baseline predictors that were not evident in the SVM analysis.

Regarding male-specific factors, our model highlighted the moderate but consistent impact of paternal age. The univariate analysis indicated that younger male partners were associated with higher success rates, and the XGBoost model reflected this, though with less prominence than female age. This aligns with studies suggesting that paternal age over 40 can negatively affect assisted reproductive technology outcomes. While semen parameters like concentration and morphology were not significant in univariate analyses, sperm motility contributed modestly in the final multivariate model. This underscores its importance in fertilization, which the more sensitive XGBoost model was able to detect.

Taken together, these patterns are consistent with established clinical knowledge: female age sets the dominant trajectory, AMH and BMI recurrently recalibrate risk across multiple subgroups, and endocrine as well as semen parameters fine-tune the prediction when baseline risk is intermediate.

Existing prognostic models provide the principal comparative framework for interpreting the present work. 

McLernon et al. (2016) derived two national calculators from United Kingdom Human Fertilisation and Embryology Authority (HFEA) data, including a pretreatment model restricted to baseline characteristics; however, their primary target was the cumulative probability of live birth across up to six complete cycles, with a separate post–first-attempt model to update risk [16].

Luke et al. (2014), using Society for Assisted Reproductive Technology (SART) registry data, estimated live-birth and multiple-birth risks over the first three cycles and explicitly required treatment-phase inputs such as the number of embryos transferred [17].

Single-centre logistic models, exemplified by van Loendersloot et al., incorporated embryo morphology and laboratory/stimulation parameters and achieved only moderate discrimination (c ≈ 0.68), reflecting a later decision point and a distinct predictor set [18].

Nelson and Lawlor (2011) likewise combined baseline and treatment-specific factors using national HFEA records [19], while Leijdekkers (2019) focused on low-prognosis women and reported 18-month cumulative outcomes [20]. Against this backdrop, our contribution differs by delivering a per-cycle, first-visit, baseline-only prediction intended for immediate, centre-level counselling, rather than a cumulative multi-cycle prognosis or a calculator dependent on treatment-phase inputs.

Although the most accurate prognosis can only be fully established at the end of the procedure—when key factors such as endometrial development and embryo quality are known—it is of immense clinical significance to establish a highly effective estimation at the conclusion of the diagnostic phase. Such a pre-procedural evaluation and predictive model serves as a crucial tool, providing couples with essential guidance on their reproductive journey and enabling more personalised treatment design from the outset.

Our study has limitations that must be acknowledged. Firstly, its retrospective, single-centre design necessitates further validation with larger, multi-centre cohorts to ensure generalizability. IVF success rates may vary across institutions due to differences in local protocols, technology, and expertise. Therefore, clinic-specific calibration of predictions, incorporating historical institutional success rates, is recommended to apply a baseline correction to the model’s estimates for practical use.

In this study, successful IVF cycles correlated significantly with younger female and male age, higher AMH, and lower FSH levels. Our XGBoost model demonstrated that even variables without strong individual significance can influence outcomes through complex interactions. By providing a reliable estimation of the expected outcome before treatment begins, a robust predictive model can significantly enhance patient cooperation, psychological preparedness, and shared decision-making, allowing for a more personalised and strategic IVF journey.

## 4. Materials and Methods

### 4.1. Study Population

A retrospective cohort study was carried out at the Institute of Reproductive Medicine, University of Szeged, based on single-centre data collected between 21 January 2022, and 12 December 2023. The primary dataset included 1243 IVF/ICSI cycles from women undergoing in vitro fertilization treatment, with or without intracytoplasmic sperm injection (ICSI), in whom successful oocyte retrieval was achieved. No further sample size estimation was conducted.

For independent same-centre external validation, an additional cohort comprising 92 IVF/ICSI cycles was subsequently collected between January and March 2025. These cases were recorded using identical inclusion criteria, laboratory protocols, and variable definitions to ensure full compatibility and standardization with the original dataset.

### 4.2. Dataset and Preprocessing

The dataset included 1422 patient observations and 15 variables; missing values were handled through listwise deletion to ensure data consistency. The target variable was a binary factor indicating IVF success (“Successful” or “Unsuccessful”). Clinical pregnancy was defined according to the International Committee for Monitoring Assisted Reproductive Technology guidelines as the transvaginal ultrasound visualization of one or more gestational sacs at 7 weeks of gestation, including ectopic pregnancies [21]. Predictor variables included demographic data (maternal and paternal age), clinical metrics (infertility duration, BMI), laboratory results (AMH, FSH, LH), and sperm parameters (concentration and motility). The dataset was randomly split into a training set (80%) and a test set (20%) using stratified sampling to preserve the original class distribution of the target variable.

### 4.3. Univariate Test of Variables

Prior to multivariable modelling, each predictor’s crude association with IVF success was examined individually. The distributional characteristics of continuous variables were assessed visually with histograms and Q-Q plots and formally with the Shapiro–Wilk normality test. As none of these variables conformed to a Gaussian distribution (Shapiro–Wilk *p* < 0.05 in all cases), non-parametric statistical methods were applied. Group comparisons for continuous variables were conducted using the two-sided Wilcoxon rank-sum test, with results expressed as medians and interquartile ranges. Categorical predictors were summarized as counts and percentages and evaluated with Pearson’s chi-squared test. A two-tailed significance threshold of α = 0.05 was used for all analyses.

### 4.4. Balancing the Training Set with SMOTE

To address the inherent class imbalance where the “Successful” class was underrepresented, the Synthetic Minority Oversampling Technique (SMOTE) was applied. SMOTE works by generating synthetic instances of the minority class by interpolating between existing minority samples. This technique was applied exclusively to the training set to improve the model’s ability to generalize and to prevent overfitting to the majority class, thereby ensuring an unbiased evaluation on the untouched test set.

### 4.5. Model Construction

**Figure 4:**
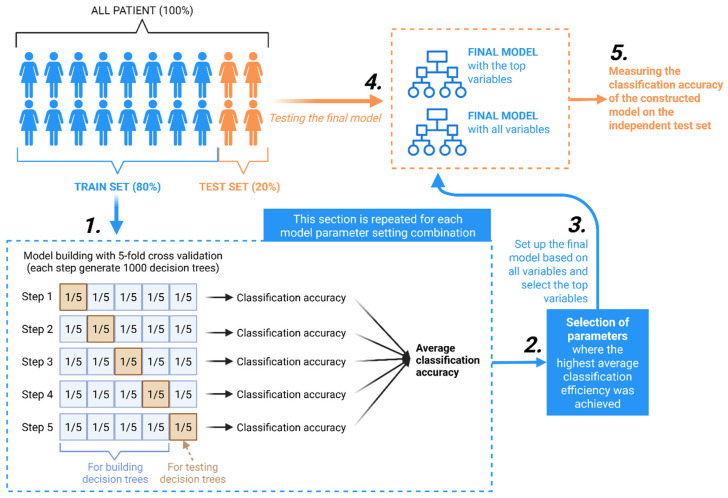
A schematic flowchart of model building. The schematic workflow of model construction illustrates the decomposition of data into training and test sets, the optimization of model parameters via 5-fold cross-validation, the construction of final models with optimal settings, and the evaluation of their efficiency on independent test sets.

A classification model was built using XGBoost, an ensemble learning method based on decision trees. The schematic workflow is summarized in Figure 4. XGBoost operates by sequentially building decision trees, where each new tree corrects the errors of the previous ones. To ensure optimal performance and prevent overfitting, the model’s hyperparameters were tuned using a 5-fold cross-validation strategy on the training set. The optimization process aimed to maximize the area under the receiver operating characteristic (ROC) curve (AUC). Key optimized parameters included the number of boosting iterations (1000), maximum tree depth (3–8), learning rate (0.01–0.3), the fraction of predictors sampled per tree (80%), and the proportion of training data sampled per iteration (80%).

### 4.6. Feature Selection and Feature Importance Analysis

To interpret the model and identify key predictors, feature importance was calculated using the Gain metric from the trained XGBoost model. Gain measures the relative contribution of a feature to the model’s performance by quantifying the improvement in accuracy brought by its splits. To further characterize each predictor’s role, we also calculated Cover, which represents the proportion of data instances affected by a feature’s splits, and Frequency, which measures how often a feature is used across all trees in the ensemble.

### 4.7. Model Evaluation

The final model’s predictive performance was assessed on the independent test set. Model discrimination was quantified using the AUC. We also calculated sensitivity (the model’s ability to identify successful outcomes), specificity (its ability to identify unsuccessful outcomes), overall accuracy, PPV, and NPV. To confirm that the model’s performance was significantly better than chance, we compared its accuracy to the NIR, which represents the accuracy achievable by always predicting the majority class. All analyses were conducted in R (version 4.5.0) using the caret, DMwR, xgboost, and ggplot2 packages.

To further evaluate model robustness, an additional same-centre external validation was conducted as the final step of testing. The locked nine-variable model (described in Section 2.3) was applied to a non-overlapping cohort of patients recruited after the original dataset had been closed. All model parameters, thresholds, and preprocessing transformations were kept fixed as determined during training and internal validation; no re-fitting or re-calibration was performed. Model predictions were then compared with observed IVF outcomes to generate a confusion matrix and derive standard performance metrics, including accuracy, sensitivity, specificity, positive predictive value, and negative predictive value. This independent assessment was designed to examine the reproducibility and clinical transportability of the model within the same institutional environment.

## Figures and Tables

**Table 1:** Description of available variables by IVF outcomes. AMH: anti-Müllerian hormone; FSH: follicle-stimulating hormone; LH: luteinizing hormone; BMI: body mass index; IVF: in vitro fertilization; IQR: interquartile range; std: standard deviation; Med: median; Min: minimum; Max: maximum; *p*: *p*-value).

Variable	Metrics	IVF—Unsuccessful	IVF—Successful	Total	*p*
Female age (years)	Min/Max	19.0/47.0	20.0/44.0	19.0/47.0	<0.0001
	Med [IQR]	37.0 [33.0; 41.0]	34.0 [31.0; 37.0]	36.0 [32.0; 40.0]	
	Mean (SD)	36.6 (5.1)	33.9 (4.4)	35.8 (5.1)	
AMH (pmol/L)	Min/Max	0.01/17.0	0.1/17.0	0.01/17.0	<0.0001
	Med [IQR]	1.6 [0.8; 2.8]	2.1 [1.3; 3.8]	1.8 [0.9; 3.3]	
	Mean (SD)	2.2 (2.2)	2.9 (2.4)	2.5 (2.3)	
FSH (IU/L)	Min/Max	0.3/26.3	0.3/31.1	0.3/31.1	<0.0001
	Med [IQR]	7.4 [6.0; 9.4]	6.7 [5.4; 8.2]	7.2 [5.8; 8.9]	
	Mean (SD)	8.1 (3.3)	7.1 (2.6)	7.8 (3.0)	
LH (IU/L)	Min/Max	0.1/28.0	0.1/22.6	0.1/28.0	0.216
	Med [IQR]	5.7 [4.2; 7.3]	5.4 [4.1; 7.2]	5.6 [4.2; 7.3]	
	Mean (SD)	6.0 (2.6)	5.9 (2.8)	6.0 (2.7)	
BMI (kg/m^2^)	Min/Max	16.6/46.6	11.2/44.1	11.2/46.6	0.491
	Med [IQR]	23.7 [21.2; 27.5]	23.7 [20.6; 27.9]	23.7 [21.0; 27.7]	
	Mean (SD)	24.9 (5.1)	24.7 (5.2)	24.8 (5.1)	
Infertility duration (years)	Min/Max	0.5/22.0	0.5/15.0	0.5/22.0	0.095
	Med [IQR]	4.0 [2.0; 6.5]	3.0 [2.0; 5.0]	4.0 [2.0; 6.0]	
	Mean (SD)	4.5 (3.2)	4.3 (2.8)	4.4 (3.1)	
Number of births	Min/Max	0/3.0	0/3.0	0/3.0	0.890
	Med [IQR]	0 [0; 0]	0 [0; 0]	0 [0; 0]	
	Mean (SD)	0.2 (0.5)	0.2 (0.5)	0.2 (0.5)	
Male age (years)	Min/Max	24.0/60.0	21.0/60.0	21.0/60.0	<0.0001
	Med [IQR]	39.0 [35.0; 44.0]	37.0 [34.0; 43.0]	38.0 [34.0; 43.0]	
	Mean (SD)	39.2 (6.3)	37.4 (5.8)	38.6 (6.2)	
Sperm concentration (×10^6^/mL)	Min/Max	0.02/250.0	0.02/250.0	0.02/250.0	0.252
	Med [IQR]	40.0 [12.0; 70.0]	40.0 [14.0; 72.0]	40.0 [12.0; 70.0]	
	Mean (SD)	46.6 (41.5)	49.5 (42.7)	47.5 (41.9)	
Sperm motility (%)	Min/Max	1.0/90.0	0.0/90.0	0.0/90.0	0.188
	Med [IQR]	45.0 [30.0; 55.0]	45.0 [30.0; 60.0]	45.0 [30.0; 55.0]	
	Mean (SD)	42.7 (17.3)	44.0 (17.2)	43.1 (17.3)	
Normozoospermia (*n*; %)	Yes (%)	393 (66.95%)	194 (33.05%)	587 (41.87%)	0.150
Asthenozoospermia (*n*; %)	Yes (%)	460 (69.70%)	200 (30.30%)	660 (47.88%)	0.618
Teratozoospermia (*n*; %)	Yes (%)	352 (68.48%)	162 (31.52%)	514 (36.66%)	0.729
Cryptozoospermia (*n*; %)	Yes (%)	67 (69.79%)	29 (30.21%)	96 (6.85%)	0.870

## Data Availability

The datasets used and/or analysed during the current study are available from the corresponding author on reasonable request.

## Abbreviations

The following abbreviations are used in this manuscript:

AMH	Anti-Müllerian Hormone
AUC	Area Under the Receiver Operating Characteristic Curve
BMI	Body Mass Index
CI	Confidence Interval
DMwR	Data Mining with R
FSH	Follicle-Stimulating Hormone
HUN-REN	Hungarian Research Network
ICSI	Intracytoplasmic Sperm Injection
IQR	Interquartile Range
IU/L	International Units per Liter
IVF	In Vitro Fertilization
LH	Luteinizing Hormone
Med	Median
Min/Max	Minimum/Maximum
ML	Machine Learning
NIR	No-Information Rate
NNGYK	National Public Health and Pharmacy Center
NPV	Negative Predictive Value
*p*	*p*-value
PPV	Positive Predictive Value
R	R (programming language)
ROC	Receiver Operating Characteristic
SD	Standard Deviation
SMOTE	Synthetic Minority Oversampling Technique
SVM	Support Vector Machine
XGBoost	Extreme Gradient Boosting
